# Air Gap Fiber Bragg Grating for Simultaneous Strain and Temperature Measurement

**DOI:** 10.3390/mi15010140

**Published:** 2024-01-16

**Authors:** Fuling Yang, Kehui Zhu, Xiaoyi Yu, Tianze Liu, Ke Lu, Zelong Wang, Yan Li

**Affiliations:** School of Mechanical and Electrical Engineering, China University of Mining and Technology, Beijing 100083, China

**Keywords:** optical fiber sensors, strain measurement, temperature measurement, Fabry–Perot interference, fiber Bragg grating

## Abstract

We propose an air gap fiber Bragg grating (g-FBG) sensor that can measure strain and temperature simultaneously. The sensor is made by aligning two fiber Bragg gratings (FBGs), and an air gap exists between these two sub-gratings. This sensor’s architecture allows it to form a spectrum with phase-shifted fiber Bragg grating (PSFBG) spectroscopy and Fabry–Perot interference (FPI) spectroscopy. Since the sensitivity of PSFBG and FPI spectra is different for strain and temperature, it is possible to measure both strain and temperature by measuring one of the reflected dips of PSFBG and the interference dip of FPI. The experimental results show that the strain sensitivity is about 11.95 pm/με via the dip wavelength detection of FPI, and the temperature sensitivity is about 9.64 pm/°C via the dip wavelength detection of PSFBG. The g-FBG sensor demonstrates a resolution of approximately ±3.7 με within the strain range of 0 to 1000 με and about ±0.6 °C within the temperature range of 25 °C to 120 °C. The proposed g-FBG sensor, characterized by its simple structure, compact size, and cost-effectiveness, exhibits significant potential in the field of multi-parameter measurements.

## 1. Introduction

Optical fiber has attracted much attention in the field of sensor applications because of its advantages of small size, high sensitivity, and anti-electromagnetic interference [1,2,3]. In order to design sensors with low complexity, simple structures, and low cost, achieving the measurement of multiple physical quantities has been identified as a crucial research focus in the field of optical fiber sensing. The cross-sensitivity is a key issue for a fiber sensor used in multi-parameter sensing [4,5,6]. The fiber Bragg gratings (FBGs) are sensitive to temperature and strain [7]. However, it is difficult to discriminate between these variables because they simultaneously influence the Bragg wavelength [8]. To address the issue of temperature cross-sensitivity in sensors, a common approach is to employ a sensing matrix method to achieve the simultaneous measurement of temperature and strain. The designed sensor typically consists of two different optical fiber structures with different sensitivities to physical quantities. Simultaneous measurement is achieved by detecting the wavelength shifts in each structure. A great deal of effort has been devoted and several approaches have been demonstrated for simultaneous strain and temperature measurement. Dan Su et al. [9] proposed a measurement scheme for dual-parameter measurements using a double-fiber grating written by a single-mode fiber and a thin-core fiber. Furthermore, there are several other approaches such as polarization-maintaining few-mode Bragg gratings [10], sawtooth stressor-assisted highly birefringent FBG [11], cascaded long-period fiber grating or Bragg grating [12,13], multimode fiber chirped long-period grating [14], superstructure FBG [15], misaligning splicing a thin core fiber between two SMFs [16], combining few-mode fiber and FBG [17,18], combining FBG and multimode fiber [19], tilted FBG [20], specially packaged FBG [21], π-phase-shifted FBG [22], and a combination of different types of interferometers and FBG [23,24,25,26]. However, there are some flaws such as relatively high complexity, difficult fabrication process, or low sensitivity in some of those approaches, which restrict their practical applications.

X. P. Dong et al. [27] proposed a novel phase-shifted fiber Bragg grating (PSFBG), which reacts when the air gap of two fiber grating changes, and can be used for strain measurement. However, the Fabry–Perot interference (FPI) between the facets of the two aligning fibers was overlooked. Y. H. Yang et al. found the FPI of this kind of PSFBG and designed a g-FBG-based tunable fiber laser [28]. Using the FPI and PSFBG spectra, it is possible to simultaneously demodulate the strain and the temperature. Furthermore, PSFBG has a narrower window bandwidth than that of normal FBG [29], so it provides a relatively high detection accuracy of wavelength shift.

In this paper, we demonstrate the unique spectrum of g-FBG, which combines two different types of spectra, PSFBG reflective spectrum and FPI spectrum. A g-FBG sensor is designed and tested. In the integrated sensor, the strain and the temperature are simultaneously measured via FPI dip wavelength and PSFBG dip wavelength. This proposed g-FBG sensor exhibits the advantages of relatively simple structure, compact size, and low error.

## 2. Sensor Structure and Sensing Principle

**Figure 1:**
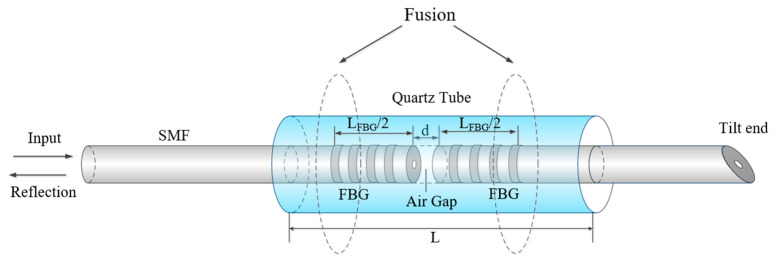
Schematic diagram of the g-FBG sensor.

**Figure 2:**
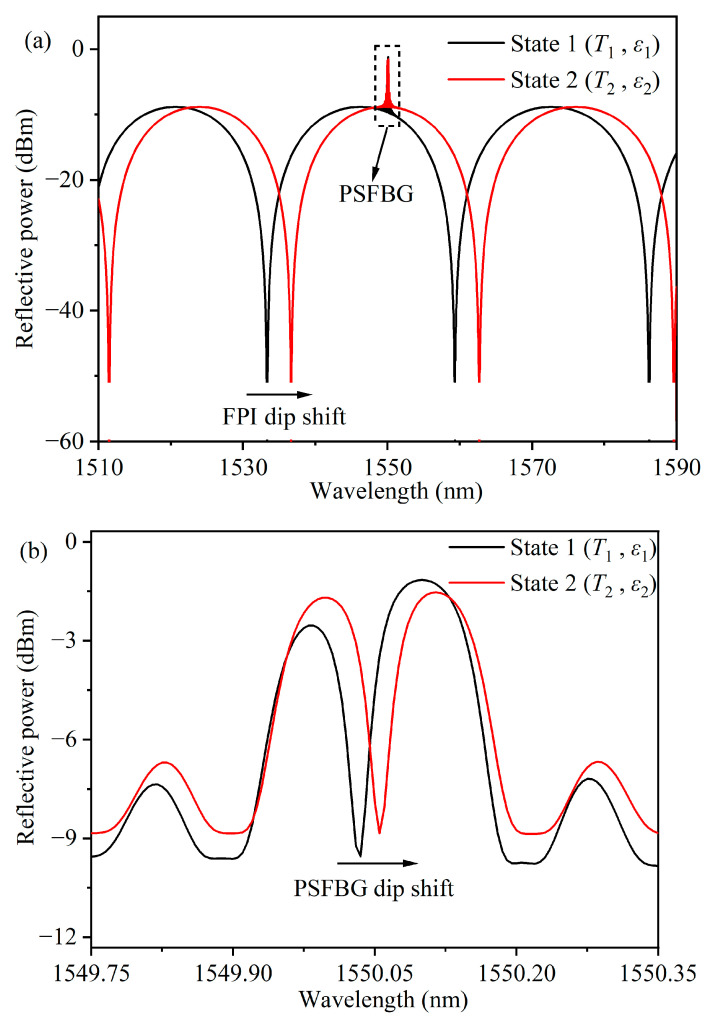
(**a**) Schematic diagram of the sensor’s reflection spectra in two different states. (**b**) Zoom out in the dash rectangle, i.e., PSFBG’s reflective spectra with different states.

Figure 1 depicts the schematic diagram of the g-FBG sensor, a fiber optic sensor. The sensor structure comprises two sub-gratings cut from the original fiber Bragg grating (FBG), positioned in the middle of a quartz tube and fixed within it through fusion splicing. An air gap exists between these two sub-gratings. Together, these two sub-gratings with air gaps constitute a PSFBG and an FPI. Fiber processing involves the use of an inclined cutting method to minimize back-reflections from the fiber-cutting surfaces. This design allows the sensor to respond to variations in strain and temperature in the environment. Monitoring the changes in the reflected spectra in two different states enables the measurement of these physical quantities. Figure 2 illustrates the schematic representations of the sensor’s reflected spectra in two distinct states.

The phase difference at the dip position in the FPI adhere to the following conditions [30]:

(1) $$\frac{4\pi n_{0}d}{\lambda_{m}}=(2m+1)\pi$$

In the given expression, *n*_0_ is the refractive index between two FBGs, and *d* is the length of the cavity. The integer *m* corresponds to the order of interference, and *λ_m_* is the central wavelength of the *m*th-order interference dip.

The calculation of the free spectral range (FSR) *S_F_* is expressed as follows:

(2) $$S_{F}\approx \frac{\lambda_{m}^{2}}{2n_{0}d}$$

For an FPI-type sensor, its spectrum of reflection linearly shifts with variations in strain (Δ*ε*) and temperature (Δ*T*). The wavelength shift Δ*λ*_FPI_ of its dip can be expressed by the following equation [31]:

(3) $$\Delta \lambda_{FPI}=K_{FPI\varepsilon }\Delta \varepsilon +K_{FPIT}\Delta T$$

where *K*_FPI*ε*_ and *K*_FPI*T*_ are the strain and temperature sensitivities of the FPI, respectively.

Like FPI, PSFBG exhibits a linear relationship between the wavelength shift of its reflection spectrum and the variations in strain (Δ*ε*) and temperature (Δ*T*). The wavelength shift Δ*λ*_PSFBG_ of its dip can be described by the following formula [22]:

(4) $$\Delta \lambda_{PSFBG}=K_{PSFBG\varepsilon }\Delta \varepsilon +K_{PSFBGT}\Delta T$$

where *K*_PSFBG*ε*_ and *K*_PSFBG*T*_ are the strain and temperature sensitivities of PSFBG, respectively.

From Equations (3) and (4), it can be seen that the wavelength shift Δ*λ*_FPI_ of FPI and Δ*λ*_PSFBG_ of PSFBG are both functions of strain change Δ*ε* and temperature change Δ*T*. As a result, when the ambient temperature and external strain change, the troughs that they correspond to shift. The wavelength shifts of the dip to which they correspond can be expressed as Equation (5):

(5) $$\left[\begin{array}{c} \Delta \lambda_{FPI}\\ \Delta \lambda_{PSFBG} \end{array}\right]=\left[\begin{array}{cc} K_{FPI\varepsilon } & K_{FPIT}\\ K_{PSFBG\varepsilon } & K_{PSFBGT} \end{array}\right]\left[\begin{array}{c} \Delta \varepsilon \\ \Delta T \end{array}\right]$$

Once the sensitivities mentioned above are determined, it is possible to derive a sensitivity matrix for the simultaneous measurement of strain and temperature through matrix transformations, expressed as Equation (6):

(6) $$\left[\begin{array}{c} \Delta \varepsilon \\ \Delta T \end{array}\right]=\frac{1}{M}\left[\begin{array}{cc} {-K}_{PSFBGT} & K_{FPIT}\\ K_{PSFBG\varepsilon } & -K_{FPI\varepsilon } \end{array}\right]\left[\begin{array}{c} \Delta \lambda_{FPI}\\ \Delta \lambda_{PSFBG} \end{array}\right]$$

where *M = K*_PSFBG*ε*_*K*_FPI*T*_ *− K*_FPI*ε*_*K*_PSFBG*T*_ is the determinant of the coefficient matrix.

## 3. Experimental Results and Discussion

### 3.1. Experimental System

**Figure 3:**
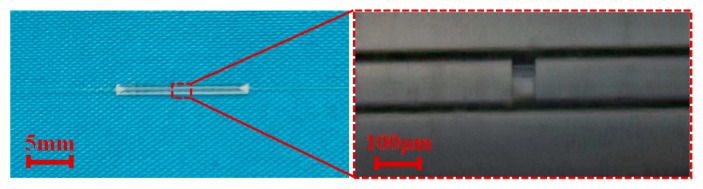
Physical picture of the g-FBG sensor and its partially enlarged image.

**Figure 4:**
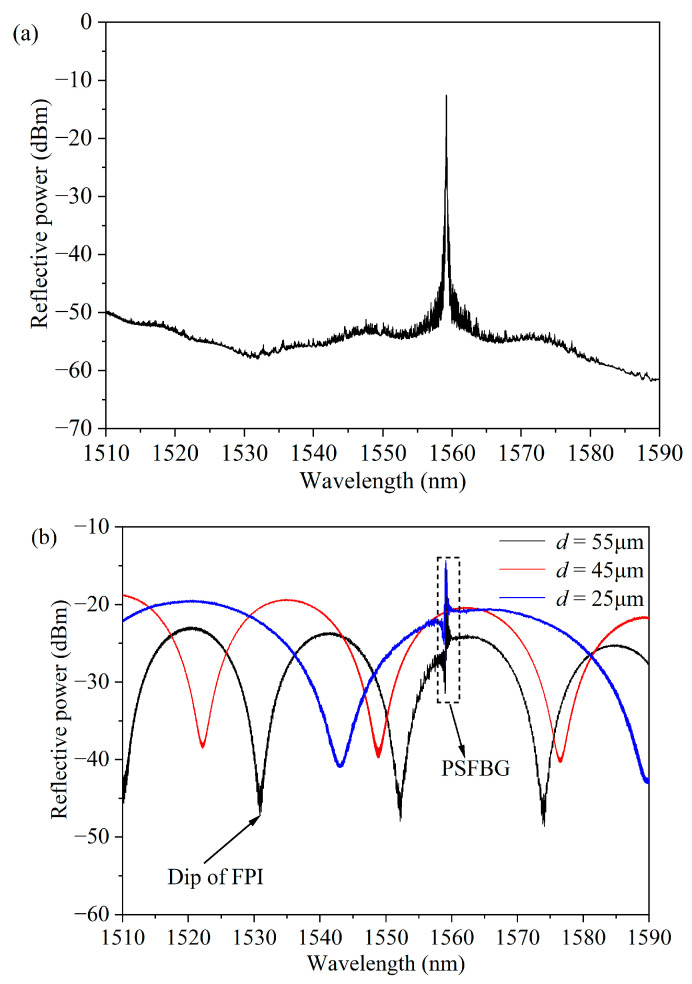
Reflection spectra of the FBG and the g-FBG sensor: (**a**) the original reflection spectrum of the FBG and (**b**) spectral diagram of the g-FBG sensor corresponding to different gap spacings.

Figure 3 shows a physical picture of the g-FBG sensor. The parameters of the sample g-FBG sensor are *L* = 15 mm and *L*_FBG_ = 10 mm, where *L* is the length of the quartz tube, *L*_FBG_ is the length of the initial FBG. The spectra of the FBG used are shown in Figure 4a, and the Bragg wavelength (*λ*_B_) of the used FBG is about 1559.376 nm. The spectrum of the g-FBG sensor under different gap spacings is shown in Figure 4b. When *d* = 55 µm, the FSR is about 21.241 nm, and the contrast is about 24.56 dB. As the gap decreases, the FSR of the FPI increases. When *d* = 25 µm, the FSR is about 46.932 nm, and the contrast is about 21.42 dB. As can be seen from Figure 4b, the dip of FPI is sharper, so the sensor with *d* = 55 µm is selected for the experiment of simultaneous temperature and strain sensing.

**Figure 5:**
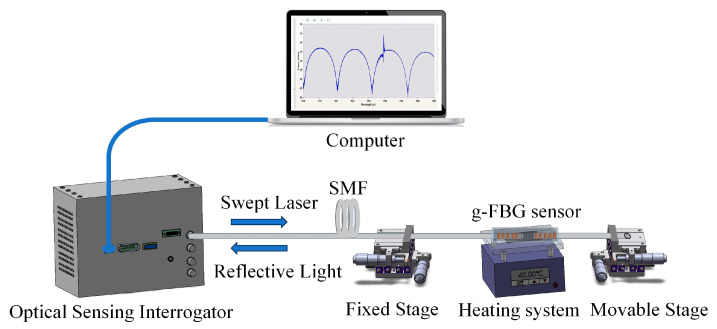
Schematic configuration of the experimental setup.

Figure 5 illustrates the schematic diagram of the experimental setup designed for the simultaneous detection of changes in strain and temperature. An optical sensing interrogator (SM125, Micron Optics, with a spectral range from 1510 nm to 1590 nm and a spectral resolution of 1 pm) is utilized to monitor the reflection spectrum of the g-FBG sensor.

**Figure 6:**
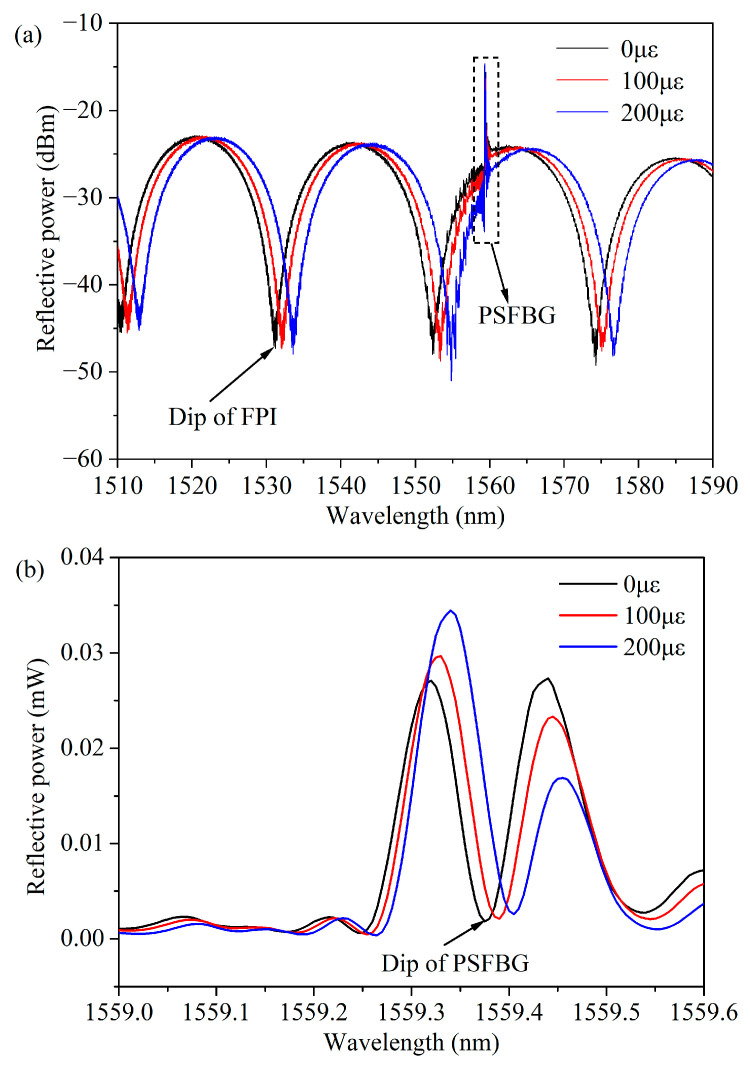
Experimental results of g-FBG sensor with different strains: (**a**) g-FBG sensor reflective spectra with different strains and (**b**) zoom in of dash rectangle, i.e., PSFBG reflective spectra of g-FBG.

The g-FBG sensor is positioned within a heating system to measure temperature characteristics. Both ends of the sensor are fixed on two longitudinal displacement platforms with an accuracy of 0.01 mm, enabling the control of the changing of the strain on the sensor. In this setup, one platform remains stationary while the other moves vertically at a low loading speed, thereby inducing axial strain on the sensor. In Figure 6, the black line illustrates the reflection spectrum of the g-FBG sensor under no applied strain at room temperature. It is observed that the edge contrast of the FPI exceeds 20 dB. Measurement indicates an FSR of 21.241 nm, and calculation using Equation (2) yields a cavity length (*d*) of approximately 55 μm.

### 3.2. Strain Measurement Test

The strain test for this sensor was conducted at room temperature (approximately 25.4 °C). The movable platform was adjusted in increments of 0.05 mm until it reached 0.5 mm, with a spacing of about 500 mm between the two fixed points. Figure 5 illustrates the partial spectrum of the g-FBG under the strain test. As the applied strain increased, both the FPI and the PSFBG exhibited a ‘red shift’ in their tilted wavelengths. However, it is noteworthy that the rate of change differed between the interference tilt of the FPI and the reflection tilt of the PSFBG.

**Figure 7:**
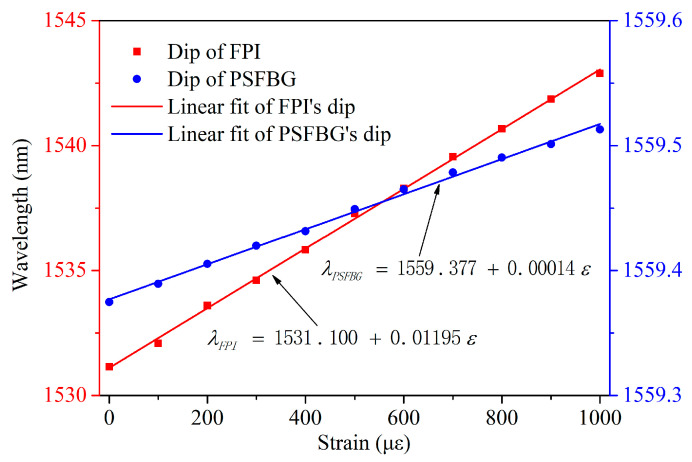
FPI and PSFBG’s dip wavelength shifts under different strains.

To analyze the reflective spectral shift of the sensor, the reflective slope of the PSFBG and the interference slope of the FPI were monitored, as depicted in Figure 6. Figure 7 illustrates the wavelength shifts of the FPI and the PSFBG as the strain increased. The PSFBG’s tilted wavelength linearly moved from 1559.375 nm to 1559.513 nm as the strain increased, corresponding to the fitting sensitivity *K*_PSFBG*ε*_ of only 0.14 pm/µε. In contrast, the FPI’s dip wavelength linearly shifted from 1531.148 nm to 1542.892 nm. Through fitting, the strain sensitivity of the FPI, *K*_FPI*ε*_*,* was determined to be 11.95 pm/µε, approximately 85 times greater than *K*_PSFBG*ε*_.

### 3.3. Temperature Test

**Figure 8:**
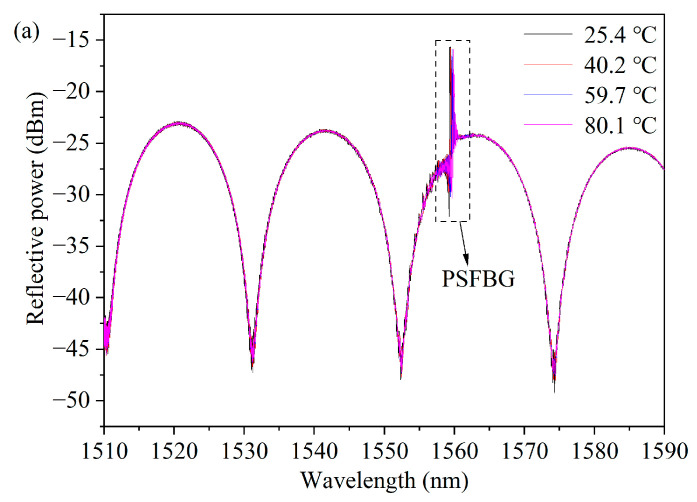

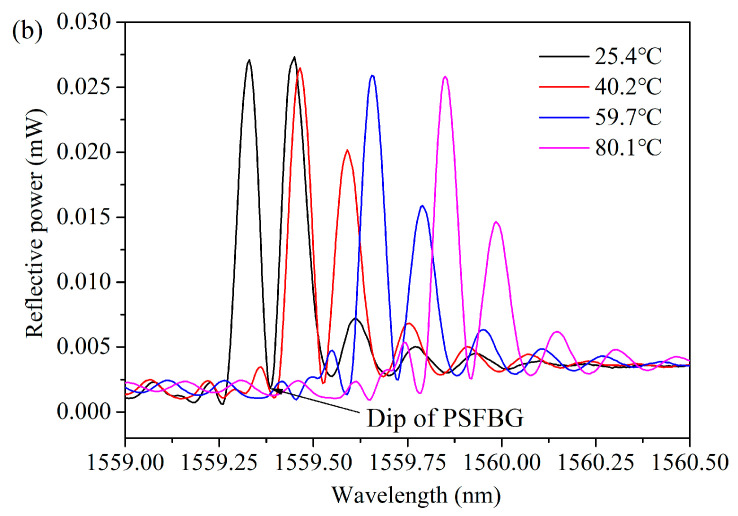
Experimental results of g-FBG sensor with different temperatures: (**a**) g-FBG sensor reflective spectra with different temperatures and (**b**) zoom in of dash rectangle, i.e., PSFBG reflective spectra of g-FBG.

The temperature response of the g-FBG sensor is investigated by means of placing it into a heating system. The precise temperature is measured by a temperature monitor. The temperature at the heating system rises gradually from 30 to 120 °C with a step of approximately 10 °C and maintains about 30 min during each temperature point. The parts of g-FBG’s spectra for different temperatures are shown in Figure 8. Because the thermal expansions of FBGs and the quartz tube are almost equal, the gap length thermal change is relatively insignificant. Thus, the FPI dip wavelength shift is relatively insignificant.

**Figure 9:**
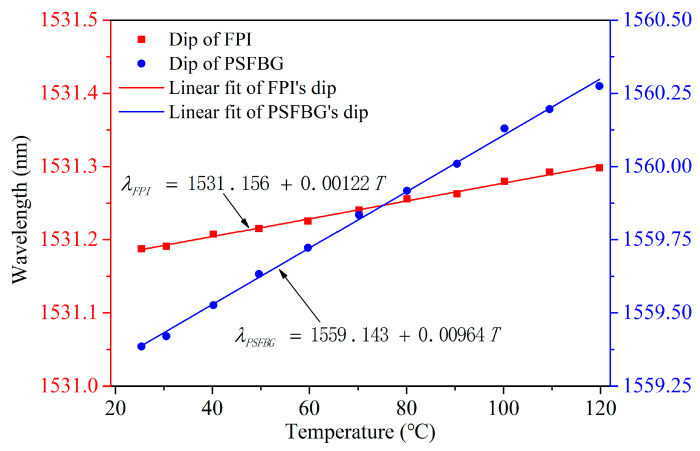
FPI and PSFBG’s dip wavelength shifts under different temperatures.

As shown in Figure 8b, the reflective dip wavelength of PSFBG shifts toward a longer wavelength when temperature rises. The measurement resolution can be relatively high owing to a narrow bandwidth (~58 pm@3 dB) of the reflective dip of PSFBG. Figure 9 illustrates the trend of the center wavelength of the dip as a function of temperature. The center wavelength of the dip shifts slightly from 1531.187 nm to 1531.298 nm, with a corresponding temperature sensitivity *K*_FPI*T*_ of only 1.22 pm/°C. Simultaneously, the center wavelength of the peak linearly shifts from 1559.385 nm to 1560.275 nm, with a corresponding temperature sensitivity *K*_PSFBG*T*_ of 9.64 pm/°C, approximately eight times greater than *K*_FPI*T*_.

### 3.4. Simultaneous Measurement of Strain and Temperature

To concurrently measure strain and temperature, we utilize the obtained strain and temperature sensitivities and apply them in Equation (6).

(7) $$\left[\begin{array}{c} \Delta \varepsilon \\ \Delta T \end{array}\right]=\frac{1}{-115.03}\left[\begin{array}{cc} -9.64 & 1.22\\ 0.14 & -11.95 \end{array}\right]\left[\begin{array}{c} \Delta \lambda_{FPI}\\ \Delta \lambda_{PSFBG} \end{array}\right]$$

Hence, in accordance with Equation (7), by measuring the reflected dip of PSFBG and the interference dip of FPI, it is possible to simultaneously determine both strain and temperature.

**Figure 10:**
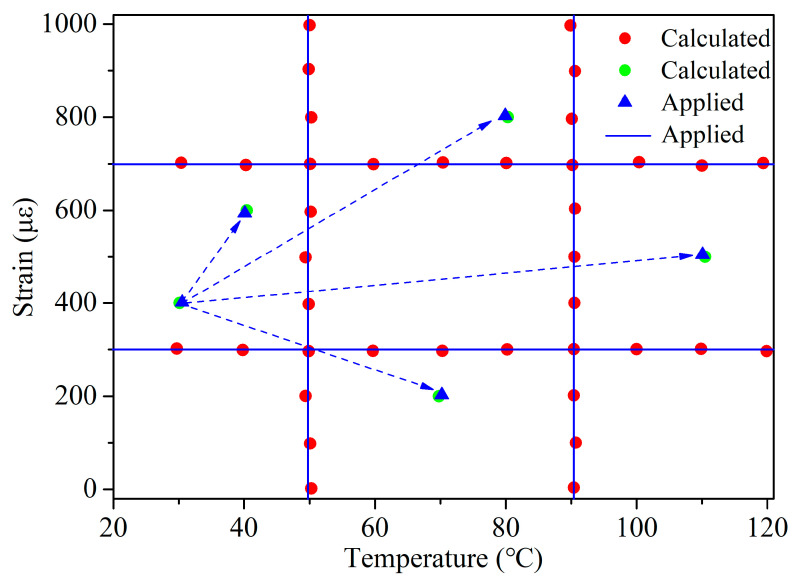
Experimental results of simultaneous measurement of strain and temperature.

To validate the stability of the sensor, two experiments were conducted. Firstly, under constant temperature conditions, strain was continuously applied to the sensor; then, under constant strain conditions, temperature was continuously applied to the sensor. The red points and blue lines in Figure 10 represent the calculated and applied values, respectively. Additionally, to better simulate the actual situation, synchronous measurements under a fixed initial state (30 °C, 400 με), of both strain and temperature, were simultaneously varied. The green and blue points in Figure 10 correspond to the calculated and applied values. It is observed that there is a minimal difference between the calculated values obtained through the sensing matrix and the actual applied values. We speculate that this difference may arise from uncertainties in measuring the applied values, such as the output of the digital temperature gauge in the heating system and the wavelengths recorded by the optical interrogator for FPI and PSFBG.

Table 1 shows a comparison of the performance of the sensor in this paper with other fiber optic sensors that measure strain and temperature simultaneously. Compared with other fiber optic sensors, the sensor proposed in this paper has advantages in terms of strain sensitivity and temperature sensitivity. The proposed g-FBG sensor may exhibit a great potential in fields requiring multi-parameter measurement due to its simple structure, cost-effectiveness, and compact size.

## 4. Conclusions

In this paper, a novel g-FBG sensor for the simultaneous measurement of strain and temperature is proposed. The performance of g-FBG sensors in strain and temperature measurements was investigated. By demodulating the two spectra of the g-FBG sensor (the PSFBG spectrum and the FPI spectrum), the simultaneous measurements of both strain and temperature can be achieved. The strain is measured with a sensitivity of 11.95 pm/με via the dip wavelength of FPI. The temperature is measured with a sensitivity of 9.64 pm/°C, ranging from 25 to 120 °C via the dip wavelength of PSFBG. The resolutions of the g-FBG sensor in measuring strain and temperature are estimated to be ±3.7 με and ±0.6 °C, respectively, in the range from 0 to 1000 με and from 25 to 120 °C. The proposed g-FBG sensor may exhibit a great potential in fields requiring multi-parameter measurement due to its simple structure, cost-effectiveness, and compact size.

## Figures and Tables

**Table 1:** Comparison of the performance of the sensor in this paper with other fiber optic sensors that measure strain and temperature simultaneously.

Configuration	Maximum Strain Sensitivity (pm/με)	Maximum Temperature Sensitivity (pm/°C)	Reference
Dual FBGs sensor	3.25	13.05	[9]
Separated micro-bend long period gratings	−2.41	8.8	[32]
Hybrid configuration of FPI/FBG	2.1	7.82	[33]
Cascaded-cavity FPIs	2.97	10.45	[34]
TCF based in-fiber MZI	−1.99	54.95	[35]
Spheroidal-cavity-overlapped FBG	3.76	8.4	[36]
Hybrid configuration of EPFI/FBG	Not mentioned	11.655	[37]
TCRF	1.08	10.4	[38]
Hybrid structure with two FPIs	5.2	13	[39]
Dual SPR effect in PCF	1.3	−6.83	[40]
TPMF	−2.37	91.84	[41]
Hybrid configuration of fiber taper/LPFG	−2.96	47.4	[42]
π-PSFBG sensor	0.7837	14.15	[22]
Femtosecond laser + FBG + DMF	1.24	9.3	[43]
Proposed	11.95	9.64	This work

## Data Availability

The data that support the findings of this study are available from the corresponding authors upon reasonable request.
